# Hydromorphone Hopes: A Qualitative Study of People Initiating Supervised Short‐Acting Injectable Opioid Treatment in Australia

**DOI:** 10.1111/dar.14092

**Published:** 2025-06-03

**Authors:** Jake Rance, Vendula Belackova, James Bell, Adrian J. Dunlop, Nadine Ezard, Marianne Jauncey, Nicholas Lintzeris, Eugenia Oviedo‐Joekes, Alison Ritter, Darren M. Roberts, Craig Rodgers, Krista J. Siefried, John Strang, Willem van den Brink, Carla Treloar

**Affiliations:** ^1^ UNSW Sydney Sydney Australia; ^2^ National Addiction Centre King's College London London UK; ^3^ Drug and Alcohol Clinical Services Research Hunter New England Health Newcastle Australia; ^4^ The University of Newcastle & Hunter Medical Research Institute Newcastle Australia; ^5^ Alcohol and Drug Service St Vincent's Hospital Sydney Sydney Australia; ^6^ Uniting MSIC Sydney Australia; ^7^ Discipline of Addiction Medicine The University of Sydney Sydney Australia; ^8^ The Langton Centre South East Sydney Local Health District Sydney Australia; ^9^ University of British Columbia Centre for Health Evaluation Vancouver Canada; ^10^ Drug Policy Modelling Program UNSW Sydney Sydney Australia; ^11^ Edith Collins Centre, Drug Health Services Royal Prince Alfred Hospital Sydney Australia; ^12^ Amsterdam UMC Amsterdam the Netherlands

**Keywords:** Australia, hydromorphone, implementation trial, qualitative study, supervised injectable opioid treatment, treatment initiation

## Abstract

**Introduction:**

A growing body of qualitative scholarship has drawn attention to aspects of supervised injectable opioid treatment (SIOT) not captured in earlier clinical trial data, identifying treatment initiation as one such area. Crucial questions surrounding people's motivations, expectations and initial experiences of SIOT remain under‐explored. This paper examines the first tranche of qualitative findings from participants of Australia's first‐ever SIOT trial, the ‘Feasibility of Opioid Injectable Treatment’ (FOpIT) study. Noting the novelty of both SIOT and hydromorphone within the Australian context, we ask: what motivated people to participate in the trial and what were their early experiences of this new treatment and medication?

**Methods:**

Fifteen semi‐structured, in‐depth qualitative interviews were conducted with participants commencing hydromorphone.

**Results:**

Almost universally, participants expressed a strong desire for change in their lives, foregrounding their intention to cease or reduce their use of street heroin. For many, the appeal of SIOT was driven by prior, unsatisfactory experiences with standard opioid agonist treatment (methadone, buprenorphine). Early accounts of hydromorphone ranged from favourable comparisons to street heroin to noting its relative shortcomings. Nonetheless, a dominant narrative emerged of reduced or ceased use of street heroin within days of beginning the trial.

**Discussion and Conclusions:**

Understanding the complexities surrounding SIOT initiation has clear implications for clinical practice, including the potential for improved treatment engagement, retention and outcomes. Accounts from FOpIT participants commencing SIOT revealed a diversity of motivations, hopes, goals and medication effects. The extent to which these are carried through to treatment outcomes is an area of ongoing research.

**Summary**

- Examines initial qualitative data from the recent Feasibility of Injectable Opioid Treatment study, Australia's first‐ever implementation trial of supervised injectable opioid treatment (SIOT).
- The newness of the medication (hydromorphone) and the novelty of the treatment administration (self‐injection) within the Australian context creates additional complexity and interest.
- Analysis focuses on two crucial questions under‐explored in the literature: what motivated people to join the SIOT trial and what were their initial experiences of treatment, including the medication itself.
- Underpinning a diversity of hopes and goals was an almost universal motivation reported by participants to cease or reduce their use of street heroin, with many drawn to SIOT due to prior, unsatisfactory experiences with standard opioid agonist treatment (methadone, buprenorphine).
- Notwithstanding the range in participant accounts of hydromorphone, from favourable comparisons to street heroin to noting its relative shortcomings, a dominant narrative emerged of reduced or ceased use of street heroin within days of commencing the trial.

## 1 Introduction

A strong evidence‐base has now been established for the clinical effectiveness of supervised injectable opioid treatment (SIOT) as a second‐line treatment for people experiencing opioid dependence for whom standard opioid agonist treatment (OAT), such as methadone or buprenorphine, has not been effective [1, 2, 3]. As research in the field has turned its attention to the challenges of implementation [4, 5, 6], crucial questions surrounding SIOT initiation remain under‐explored [7]. Initiation is, Mayer et al. [7] argue, a ‘critical point in treatment engagement that is not well‐understood in relation to these treatment options’. This paper seeks to address this gap in the literature.

Drawing on qualitative data collected from participants of the Australian ‘Feasibility of Opioid Injectable Treatment’ study (FOpIT), we examine: (i) why participants joined the trial—their initial hopes, expectations and goals? And (ii) what were their early experiences of the treatment medication, injectable hydromorphone? Adding a further layer of complexity and interest to our analysis is the time‐limited nature of the treatment and the newness and novelty of both SIOT and hydromorphone within the Australian context. How, in the absence of prior experience, did FOpIT participants come to know this new treatment and medication?

The general OAT literature emphasises the importance of initiation in shaping onward treatment trajectories [7], describing it as ‘a critical site of narrative formation’ [8] affecting subsequent retention and outcomes [9, 10]. Past research has shown that what both distinguishes SIOT from standard OAT and defines its appeal to consumers, is the free, legal and safe access to a short‐acting, injectable opioid—typically diacetylmorphine (pharmaceutical heroin) but also hydromorphone [11, 12]. It is the appeal of a more rewarding experience of treatment with these medication that underpins the success of SIOT. Such appeal is likely to matter, not only in terms of attracting people into treatment but retaining them. Treatment satisfaction, consistently high among SIOT recipients [13, 14, 15], has been identified as an indicator of both treatment retention and response [13].

A range of other motivations underpinning people's engagement with SIOT have also been noted, including improving one's health and social circumstances; avoiding trouble with the police; restoring familial relations; alleviating financial stress; achieving ‘stability’; and returning to ‘normal’ life [7, 15, 16, 17]. It is important to recognise that SIOT affords people a continuum of legitimate treatment aspirations, from reducing harm to achieving abstinence, or over time, a combination of both [16, 18]. Given its standing as a ‘second line’ treatment, people enrolling on a SIOT program have invariably attempted other treatment options, including standard OAT. People initiating SIOT tend to report high levels of motivation and readiness for change [7, 14, 16] underpinned by a conviction that (for a range of reasons) oral OAT has not worked for them [7, 19, 20]. For some, the experience of previous ‘unsuccessful’ efforts on OAT becomes the motivation to try something new, especially in settings where SIOT remains a novelty [16]. Here, in part at least, SIOT becomes attractive simply by virtue of what it is not [16].

While the extant SIOT literature affords us some valuable insights regarding treatment motivations and initiation, there is a dearth of literature in relation to the second thread of our analysis: participants' initial experiences of injectable hydromorphone. The majority of SIOT RCTs compared the use of injectable diacetylmorphine with oral methadone alone, although two Canadian studies (NAOMI and SALOME) included injectable hydromorphone as a potential treatment alternative [19, 21]. While both studies found that the latter yielded comparable results to diacetylmorphine when compared with oral methadone alone, they also noted that participants were unable to ascertain, beyond what could be expected by chance, which of the two injectable medications they had been allocated [22]. Although the literature includes some firsthand accounts of the two medications, the participants remained blinded as to which one they were describing [22].

The Canadian findings consolidated the case for hydromorphone as an alternative SIOT medication [23] in jurisdictions like Australia, where regulatory and political challenges exist regarding the use of diacetylmorphine [6, 24]. Until the advent of FOpIT, Australia has been stuck in somewhat of a political and policy impasse regarding short‐acting injectable treatment [24]. While a ‘heroin trial’ was proposed for the Australian Capital Territory in the late 1990s, the proposal faced vehement opposition by elements of the tabloid press and was ultimately vetoed by a conservative Federal Government ideologically suspicious of the initiative [20, 23, 25, 26]. In Australia, hydromorphone remains largely unknown among people who inject drugs. For FOpIT participants, this meant that not only was the treatment modality new and novel, but so too was the treatment medication. Given that much of the appeal and the effectiveness of SIOT hinges on the provision of a ‘rewarding’ medication, participants' experience of injectable hydromorphone will form an important part of evaluating the trial's overall acceptability.

## 2 Methods

FOpIT began in April 2022 via a public, outpatient OAT program run by St Vincent's Hospital, Sydney; a detailed account of the trial setting and design can be found in Rodgers et al. [1]. The primary aims of the trial were to assess the feasibility and acceptability of a time‐limited, integrated SIOT using injectable hydromorphone. Participants were offered intensive, highly‐structured treatment comprising up to twice daily, supervised injectable hydromorphone in conjunction with standard OAT. A novel feature of the trial was the time‐limited availability of hydromorphone treatment (up to 2 years) after which participants were offered maintenance on standard OAT [1]. Quantitative and qualitative interview data were collected at three timepoints during the trial. This paper analyses qualitative data collected during the first tranche of semi‐structured, in‐depth interviews held with participants shortly after commencing injectable treatment.

Following their induction onto injectable treatment, participants were introduced to a member of the University of New South Wales research team (J.R.) to schedule an initial research interview. This interview comprised a series of quantitative measures, including quality of life, mental health, and recent illicit drug use [1]. All participants were expected to participate in the collection of quantitative data. Twenty‐one of the 22 enrolled participants took part, with one participant voluntarily discontinuing treatment.

During this initial research interview, participants were offered the opportunity to take part in an additional, qualitative interview, with a maximum of 15 set by the study team. Participants were assured that all interviews were strictly confidential and anonymous, and that the researcher conducting the interviews was not part of the trial's clinical operations. All interviews were conducted by the first author (J.R.) and took place in a private room within the centre. Interviews lasted 25–85 min. All participants provided written consent. Interviews were conducted within 3 weeks of participants' first dose of injectable hydromorphone, with an average of 9 days and a range of 1–21 days. It is important to recognise that individual variability in the induction process and the timing of interviews would likely have affected participants' experiences of hydromorphone and its ‘effects’. Full details regarding the trial's hydromorphone dosing protocol can be found in Rodgers et al. [1].

Interview questions included: participant histories of opioid use and treatment; motivations for joining FOpIT; hopes, expectations and possible goals for treatment; navigating daily service delivery; sense of agency in treatment decision‐making; relationships with staff; and finally, initial experiences of injectable hydromorphone (including intravenous or intramuscular administration). Participants were reimbursed for their time and expertise with a $50AUD voucher. Interviews were digitally recorded, professionally transcribed, checked for accuracy and deidentified.

Our analysis followed the basic tenets of thematic analysis [27], combining data‐driven (inductive) and analyst‐driven (deductive) approaches. Inductive themes were identified by the first author (J.R.) during initial, close readings of the transcripts. These emergent themes, along with indicative quotes, were subsequently discussed with members of the study team, A.R. and C.T. Our combined knowledge of the extant SIOT and OAT literature, including gaps identified in the former, served as our analytical framework, guiding our discussion of inductive themes and the ultimate determination of our two research questions: treatment initiation and early hydromorphone experiences.

Aided by our knowledge of the opioid treatment literature, our analysis of the data was further informed by conceptual insights drawn from the sociological literature on alcohol and other drugs, such as Berridge's ‘futurizing history’ [28] and Hardon's ‘fluid drugs’ [29]. We also note the important contribution of the research interview itself and the opportunity it afforded participants to think‐aloud and think‐through, to clarify their thoughts, and to find, shape and account for themselves. Our final analysis, including primary themes and associated secondary themes or subheadings under which our findings are organised, reflect this combination of inductive and deductive analysis; or what Fereday and colleagues refer to as a ‘hybrid’ approach [30]. The writing process was led by J.R., including decision‐making about content, analysis and structure, and reviewed by A.R. and C.T. At critical junctures, drafts of the manuscript were distributed among the broader co‐authorship team. All feedback was reviewed by J.R., discussed with A.R., and integrated within the manuscript where appropriate.

Approval for this study was provided by St Vincent's Hospital Human Research Ethics Committee (2019/ETH00418). Pseudonyms have been used throughout the paper. Pseudonyms were chosen to loosely reflect the typically Anglo‐European names of participants. Plural pronouns have been used exclusively to ensure the anonymity of the participant identifying as non‐binary.

## 3 Results

A total of 15 participants were interviewed, ranging in age from 28 to 58 and including eight men, six women and one participant who identified as non‐binary. Two of the men and the non‐binary participant identified as gay and one as bisexual; two of the women identified as lesbian. All 15 participants reported receiving some form of social welfare support payment, with three also reporting part‐time employment and one full‐time. Two participants reported being homeless, while another faced imminent eviction. All participants reported prior periods on OAT, albeit varying considerably in duration from months to decades. Thirteen participants were receiving OAT at the time of enrolment on FOpIT, including 11 on methadone, one on sublingual buprenorphine‐naloxone (‘Simon’) and one on long‐acting injectable buprenorphine (‘Ben’). The two participants not on OAT, ‘Billie’ and ‘Jean’, were started on methadone as part of their induction on the study.

Our findings are organised into two sections. The first section examines participants' reflections on their motivations, hopes and goals following their initiation on injectable treatment; the second focuses on participant accounts of injectable hydromorphone and its effects.

### 3.1 Expectations, Hopes and Goals

#### 3.1.1 A Sense of Optimism

Offered the combination of a new pharmacotherapy and novel treatment administration, perhaps unsurprisingly, FOpIT participants tended to report high levels of confidence that this was *the* treatment most likely to ‘work’ for them. Either implied or explicit was the sense among participants that what distinguished SIOT unique from existing OAT options—*short‐acting opioid* plus *injectability*—was what would make it effective. As Ava and Alice explained: ‘This is very much a far more likely trajectory to successfully stopping using [heroin] than any other treatment that I've tried, by far. By far.’ (Ava); ‘This is perfect because it's the only way that I'm gonna be able to stop. Really, it is.’ (Alice). Ben emphasised the importance of the injectable component of FOpIT:

[Y]our brain just associates that needle with a decent high and if they did give you a tablet, you'd be thinking in your head, “How can I break this down into something I can shoot up? And how much better would it be then”.

Like most FOpIT participants, Ben had tried numerous forms of treatment over the past 20 years, from withdrawal treatment and residential rehabilitation to OAT with methadone and buprenorphine (including long‐acting injectable buprenorphine). For Ben, FOpIT ‘just seemed ideal’. As he explained, he no longer needed to ‘worry about where my next opiate's gonna come from […] there's doctors and nurses involved […] it's comforting to know that I'm in satisfactory health to go home.’

Aron too had tried multiple treatment approaches and had spent much of the past decade ‘jumping on and off’ methadone treatment. Just recently, Aron explained, he and his partner had even mooted the possibility of migrating to Switzerland in the hope of joining one of its injectable diacetylmorphine treatment programs. Aron recalled his reaction when first learning about the Sydney trial: ‘FOpIT for me was a miracle […] Free dope. Pharmaceutical grade. Clean, safe environment … [I]t just ticked all the boxes.’ Aron's described his ultimate treatment goal as abstinence from street heroin and identified SIOT as the most likely means of achieving it. His determination ‘100 per cent clean’ by the end of FOpIT was partially motivated by recognising the impact ‘his habit’ had on his partner and mother.

It's gonna give me my life back and it's gonna give my kids their father back, and it's gonna give my mum her, her son back, and my siblings their brother back. And … my partner her partner back. And that's how serious this is.

Like Aron, others were similarly motivated by concerns they held for the impact their lives were having on partners and families. As Jack noted, ‘My mum was more keen for me to get on it [FOpIT] than me […] I'd be using somewhere safe and she wouldn't have to worry.’ For Simon, his intimate relationship had been his pre‐eminent consideration when deciding to join the trial and in fashioning his hopes for success:

[I]t's a win‐win situation for me. I spoke about it with my partner. We had heaps of discussions before I came 'cause it was important for both of us. […] I'm relying on this to help me be the person I want to be, so I could be good for her too.

For these participants, and others, FOpIT afforded hope and the possibility of change: of personal, social, even existential, transformation. While such aspirations are not uncommon among people seeking treatment per se, what distinguishes them here is the conviction that SIOT is *the* pathway to realising them. For Billie, ‘it was just time … I'm getting old, too old to use, really. And just no veins … I wanted to years ago, really, but I just couldn't bring myself to just go on methadone’. Unlike most other participants, Billie had had little prior experience with OAT, other than a brief stint on methadone associated with a court matter. For Billie, injectable hydromorphone was central to the appeal and acceptability of FOpIT: ‘That's the key, isn't it? Like everyone would be alright with that 'cause it's a close relative, you know. Methadone's a long way from heroin, really. And buprenorphine is just no comparison.’ For Billie, injectable hydromorphone promised a genuine alternative to heroin; a treatment combining the facility to inject alongside the valued effects of an opioid—effects participants consistently described as conspicuously absent from oral methadone (and buprenorphine).

#### 3.1.2 A Chance of ‘Stability’

While some participant narratives incorporated the language of ‘abstinence’ (from street heroin), others emphasised ‘stability’ and the opportunity FOpIT afforded them to recalibrate their relationship to street heroin rather than necessarily cease its use. Ultimately, however, we suggest such differences are best understood as a matter of narrative emphasis and nuance rather than a simple binary. What remained a constant among participants was the overall goal of changing their heroin use to get ‘back to life’. FOpIT afforded the opportunity to escape the relentless demands of the illicit market while still granting (safe and legal) access to an injectable opioid. As Alice put it: ‘[Y]ou've got no idea what I do every morning. Six‐thirty out … The shit I do to get [heroin] … Oh, it's crazy work mate.’ Such respite, participants explained, would serve as a valuable opportunity to address aspects of their lives they identified as no longer functional, especially around their heroin use:

I just wanted to be more structured and have a bit more self‐discipline around opiates […] I'd like to get back to how … I was only using once a week and I did it for ages. Like two years, three years or something […] I stuck to it for a long time. But, yeah, I'd like to get back to that.(Jack)

Like Jack, neither Noah nor Ezra reported an interest in achieving abstinence—from heroin or any other illicit drug—but rather, as Ezra put it, by a desire ‘just to have more stability in my life.’ Ezra compared the appeal of FOpIT over methadone: ‘it's injectable and closer to heroin’. While for Nino it was ‘too early to say’ what their long‐terms plans were, SIOT nevertheless represented a ‘sensible next step’ in their treatment journey, affording not only legality but a sense of legitimacy:

It is liberating […] I always thought that if I was given the opportunity to have my injections without the legal … you know, freely, that my life would be stable and great, and it is. It redefines the whole drug‐using experience. Takes away all the illegality, all the sort of social stigma […] It becomes a treatment.

#### 3.1.3 Opportunistic Timing

While the majority of participants accounted for FOpIT's appeal in terms of its novel, injectable component, for two participants, Luna and Charlie, their interest was more opportunistic. For both, the nature of FOpIT as a trial of SIOT was of less interest than the opportunity it afforded them to try a new treatment. Indeed, Luna reported being initially unaware of what medication FOpIT was trialling. Both couched their hopes in terms familiar to most seeking drug treatment: ‘sick and tired’ of their drug use and desperate to ‘just give something else a go’.

I really wanted to just try almost anything to just stop, stop the destructiveness in my life … the day‐in, day‐out personal and psychological, and physical destruction of my life through using heroin … Like I was just so sick of it […] And then FOpIT came along.(Luna)

Given their previous experiences with a range of drug treatment options, including the previous 12 months on methadone, what was most salient for Luna was the opportunity to try something *different* and to try it *now*. Like Luna, Charlie had also been using street heroin ‘on top’ of their methadone dose prior to FOpIT. For Charlie, as with Luna, the SIOT aspect of the trial was of less interest than the possibility of change, albeit something broader, more inchoate than it was for Luna:

just to try and make things better with my life, I s'pose, and see, see if it [FOpIT] works; see if it helps […] Try to just step back and just take a whole look at my life […] just try and get my shit together […] get my tax file number fuckin' done and dusted 'cause I've been fuckin' itchin' about that […] I wanna slow down on my drinkin' too.

While Charlie reported wanting to ‘slow down on my drinkin' too’ and Luna emphasised their desperation to secure housing, the primary object of treatment for both participants was respite from their heroin use. As Luna put it, ‘I feel lucky and privileged to be able to be a part of it 'cause so far it's working.’

#### 3.1.4 Participating in the Future

A desire for individual‐level change—albeit one necessarily entangled with relational, familial, financial and social elements—was a bottom‐line motivation among nearly all participants. For some, however, the trial also afforded hope for *collective* change, for the future of SIOT in Australia and for innovation in drug treatment more broadly. Among these individuals, participation in the trial was motivated by a sense of collective responsibility and shared purpose. Keenly aware that FOpIT was Australia's first‐ever trial of SIOT, they felt a sense of responsibility for the treatment futures of others who inject drugs. Participants like Tina recognised that because FOpIT was ‘the first of its kind in Australia, it means we should do a good job, so there's a future for it’. Participants reporting feeling that they were ‘part of a bigger thing’ (Ava) and wanting to ‘make it work for other people’ (Jack).

As participants attempted to contextualise and account for their own experience of FOpIT, their personal narratives inevitably entangled with broader national and global narratives of drug treatment, its histories *and* its futures. Billie, for example, referenced the contemporary appeal of FOpIT in terms of Australia's failed attempt to implement a ‘heroin trial’ in the 1990s. Recalling the disappointment and abandonment of hope that followed the latter, Billie described their response to discovering a new trial was finally underway:

Amazing … like it's just the best thing I could imagine. Like we thought this was gonna happen like 20 years ago and it was so disappointing that it didn't […] So, people will be amazed if they ever find out about [FOpIT] It will just change their lives, wouldn't it?

Participants' widespread enthusiasm for the trial—from feeling ‘special’ (Ezra) and ‘proud’ (Jean) to ‘ground‐breaking’ (Aron) and incredulous: ‘I still couldn't believe it’ (Billie); ‘it blew my mind’ (Simon)—reflected the historical absence of SIOT as a treatment option in Australia. Ava's gratitude for FOpIT included a more explicit reference to the contested nature of Australian drug treatment policy:

how touched I am and how appreciative I am that there's people who are fighting for change which is so desperately needed. People who are allies […] Like these are incremental, tiny, tiny, little steps on that new path, but it's kind of like how you crack a windscreen: you just need a tiny, little crack and then, once that goes, it weakens the whole.(Ava)

Participants were often aware that SIOT programs have been operating successfully overseas, drawing on this knowledge to explain FOpIT to their peers—‘It's like the heroin injection trial that they do overseas’ (Jean)—or to reflect critically on drug treatment in Australia: ‘how antiquated our approach is and how new shit has got to be tried’ (Ava). For some participants, FOpIT was simply one treatment option of many that should be available: ‘What I would like to see is as many treatments available as possible’ (Tina); ‘I feel like something else is needed because methadone and bupe; it's not enough […] There's countries that have got like six, seven, eight options’ (Jean). For Jean, this was even more reason to ensure that FOpIT succeeds:

I really think there's a lot of people that this could help. Every single person I've told about it is like, ‘Fuck off! Is that even real?’ Like, yeah, they never thought anything like this would happen in Australia.

And Jean, like others, was determined to be part of that future: ‘Like in 30 years [I can say] I was one of the first people on the hydromorphone. I helped make it happen.’

### 3.2 Experiencing Injectable Hydromorphone

#### 3.2.1 Initial Impressions

Although several participants were familiar with ‘Dilaudid’, the trade name for the shorter‐acting version of hydromorphone, only one participant reported any previous experience with hydromorphone. Nevertheless, participants reported extensive experiences of other opioid use, primarily heroin, and it was through these embodied histories that their initial impressions of injectable hydromorphone were mediated and translated. Alice, for example, described the experience of injectable hydromorphone as ‘the same as having a shot [of heroin]. Like it's exactly the same’. Others, like Simon, described the experience as ‘remarkably similar’ to using heroin, the ‘taste’ and the ‘indicators that you look for in satisfaction’; or Nino: ‘it goes to the same place but it's just a little bit different. Not inferior.’ Having started hydromorphone treatment, Aron, like others, was curious to compare it heroin:

I've had two really small shots [of heroin] since I've been in FOpIT […] And, and I just went, “Fuckin', I don't fuckin' need it, man. This other shit's fuckin' as good, if not better.” I wouldn't say better but as good.

Several participants characterised their initial exposure to hydromorphone as having *no* effect. Likely this reflected a combination of factors, including levels of pre‐existing tolerance to opioids and how far along the induction process they were at the time of interview. As Tina, for example, put it: ‘I haven't had enough of it to do anything’. Billie similarly reported that ‘I haven't actually felt it yet’, before elaborating: ‘Well, I don't feel the methadone either [laughter]. I haven't felt anything yet. Like it's only been 10 days. I'm on 50 mg of methadone, so I didn't think I would feel it.’ Jean, however, was less equivocal in her criticism: ‘I wouldn't compare it to heroin in any way, except for you inject it.’ Nevertheless, despite being ‘convinced they're giving me water […] I haven't felt anything yet!’, Jean ‘hoped it would get better’.

It is important to contextualise these reports of ‘no effects’. Tina's initial, sporadic attendance hampered their opportunity for an increase in hydromorphone beyond the modest starting dose of 10 mg (as per the FOpIT protocol). Billie and Jean were the only two participants not on OAT prior to FOpIT and as such required inducting on to methadone several days prior to their first dose of hydromorphone. Jean was interviewed just 2 days after commencing injectable treatment and as such was effectively on starting doses of both methadone and hydromorphone. Billie reported a longstanding and significant heroin habit, and as such it was not surprising that they reported feeling no effect from either medication at this early stage in the trial. In terms of administration, we note that Tina and Billie tended to inject intramuscularly, while Jean did so intravenously.

Other participants referred to a process of adjustment, of *getting to know* the drug: ‘my body's recognising it now’ (Nino); ‘I'm becoming accustomed to it’ (Noah). As Nino elaborated: ‘I'm doing it IM [intramuscularly] and what I've found is that my body's become used to it […] I can feel it before I get the needle out, which is amazing. I can feel it. I can taste it.’ Here, Nino's account resonates with recent neurobiological research on the physiological effects of ‘expectation’, described in both the alcohol and stimulant literature [31]. Both Nino and Noah's observations also echo what French sociologist Emile Gomart [32] has described as a period of ‘apprenticeship’ in which bodies come to recognise and learn to appreciate specific drugs or pharmacotherapies.

#### 3.2.2 The Making of ‘Effects’

Perhaps inevitably in a jurisdiction where heroin remains the most frequently injected opioid and more potent opioids such as fentanyl are still relatively uncommon [19], heroin became the benchmark through and against which participants mediated, evaluated, and qualified their experiences of hydromorphone. One such qualification was the distinction participants drew between its immediate effects and those that occurred hours later. This distinction was relatively common among participants, whether they administered hydromorphone intravenously or intramuscularly. For example, Ezra (injecting intravenously), explained that while ‘you don't get that warm sort of rush [but] afterwards, I've noticed … I can relax and get a bit of a nod’. Similarly, Ava (injecting intramuscularly), while reporting minimal immediate effects—‘would I call it a rush? No way. Like not even by a longshot’—nevertheless suggested that ‘the stone’ they experienced for several hours later was ‘surprisingly’ similar to heroin: ‘So, if we disregard the initial period, right, and just assume that the drugs have taken effect, I would say it's better than the heroin.’ This absence of an initial ‘rush’ followed by a ‘longevity’ (Ben) of effect was commonly observed regardless of participants' route of administration.

The impossibility of detaching the effects of a drug from the setting in which they occur was implicitly acknowledged by participants as they differentiated between their experiences of opioids both inside and outside the trial setting. Oliver, for example, described his longstanding attachment to what he referred to as ‘doctoring’—the ritualised aspect of injecting, such as the mixing up the drug solution in a spoon and the drawing it into the syringe—which was no longer possible within the trial. ‘It's a ritual kind of thing that I like to do which I can't do here’, he explained, ‘which throws it [hydromorphone] so it's not similar to heroin because it seems like it's a clinical, other drug that I'm having here.’ Other participants noted the way in which the effects of hydromorphone itself seemed to materialise differently depending on the setting, particularly when combined with other substances:

So, doing this in a straight environment where there's no tobacco, no things to help it [hydromorphone] kick in … that's the only thing that's different. (Simon)

But I s'pose like, 'cause I smoke cones as well like when I get home and have a beer or something, and it makes you a bit cruisy. It's nice. It's nice and cruisy feeling. Not right then and there when you have it but then, when you get home.(Charlie)

#### 3.2.3 Negotiating Initial Concerns

While participants reported a range of hydromorphone‐related effects, such as the ‘morphine itch’, they also raised broader concerns regarding the way the trial protocol governed its use. Several participants challenged the trial's stipulation that only arms were permitted for the administration of hydromorphone. For those accustomed to accessing veins elsewhere on their body, such as neck or groin, ‘muscling it’ became their only option. While some, like Nino, accepted this was a non‐negotiable condition of the trial—‘I would love to do it IV, but [because I cannot access a vein in my arm] I accept the IM and it's lovely’—others registered their disappointment:

Well, that's what it's about, isn't it? It's about IV users. […] And [staff] go, ‘Oh, no, but in Canada they did it.’ I said, ‘I don't give a fuck. You're doing it here in Australia. We're IV users. We're not fucking injecting into our muscle.’ […] I mean I can get a vein like that [clicks fingers] but it's not where they will legally let me go, so …(Tina)

Several participants were similarly opposed to the trial requirement that all participants receive a long‐acting oral OAT in conjunction with their hydromorphone. While acknowledging the short‐acting nature of hydromorphone, they nonetheless argued that oral OAT should be optional and a matter of individual choice. Indeed, before becoming aware of this requirement, several participants had initially envisaged FOpIT as a means of ‘coming off’ their methadone. As Jack explained:

I thought, initially, that [FOpIT] was just this [hydromorphone], not methadone. I thought I could [use FOpIT as a way to] get off methadone a lot easier […] It was a massive part [of my motivation]. It was probably the biggest part, yeah … When I found out you've gotta be on methadone as well, I thought, “what's the fuckin' point?”

Despite registering these concerns, both Jack and Ava nonetheless noted with genuine surprise their diminished interest in heroin. Ava, for example, while finding her intramuscular injection of hydromorphone ‘hurts like hell’, nevertheless recounted: ‘I can't tell you how bizarre it is that I haven't gone to get on for two days […] I never had that with methadone or bupe.’ Similarly, Jack, despite his disappointment with hydromorphone—‘I thought it was gonna be very close to heroin […] But it's not, it's nothing like gear’—acknowledged that he had stopped using heroin since starting the trial: ‘Something snapped in me. Something's changed […] I don't know what's going on with me. Maybe I'm maturing. I don't know.’ Other participants, like Ben, were similarly surprised by how quickly their relationship with heroin seemed to shift: ‘I haven't used [heroin] once since I started last Monday. It's fantastic.’

## 4 Discussion

A defining feature of SIOT and a key to its effectiveness lies in offering a more rewarding medication to attract and retain people in structured treatment whilst simultaneously creating an opportunity to effect other changes in lifestyle and well‐being. Our findings appear to support this core principle, with the attraction of a short‐acting, injectable opioid seemingly effective in motivating FOpIT participants to initiate treatment. In keeping with previous findings [7, 16, 33], the sense of frustration and futility participants associated with previous experiences of oral OAT, coupled with a desire and a readiness for change, appeared to have fuelled their sense of hope and expectation for a new and novel treatment.

It is important to acknowledge, however, that the appeal of something ‘new and novel’ alone does not explain the specific appeal of SIOT. Witness, for example, the recent popularity surrounding the introduction of long‐acting injectable buprenorphine, characterised by some as ‘a game changer’ [34]. We contend that beyond the appeal of newness and novelty, it is the core elements of SIOT which underpinned participants' sense of shock and disbelief that such a ‘ground‐breaking’ trial was taking place in Australia. For FOpIT participants, SIOT offered a treatment approach and a medication that not only acknowledged the sought‐after effects of street heroin relative to existing OAT options, but for many, their deeply‐held attachment to the practice of injecting. Perhaps participant Ezra put it best when he described the appeal of FOpIT as ‘injectable and closer to heroin’.

Among trial participants, motivations to initiate treatment ranged from a sense of desperation to try something new, to cogent arguments as to why SIOT was *the* treatment option most likely to ‘work’ for them. Some participants were also motivated by a sense of altruism and collective responsibility, describing their determination for FOpIT to succeed so others could also benefit from the same opportunity. We note this sense of shared purpose and responsibility for collective change is similarly evident in clinical trials, especially in cancer research (e.g., [35]). While some treatment narratives were orientated around a primary goal of ceasing or ‘abstaining’ from illicit heroin use, others were organised around a desire to regain ‘stability’ and ‘structure’ vis‐à‐vis their long‐term heroin use. Irrespective of their intentions, the respite from the demands of the illicit market afforded participants a therapeutic hiatus, a chance to review and perhaps revise their initial intentions—an opportunity described elsewhere as ‘a chance to stop and breathe’ [15]. For some participants, their treatment goals incorporated a desire for change more broadly: in relation to housing, family relationships, intimate partnerships, money and a sense of (legal) legitimacy.

The relationship between treatment sought and treatment received is more complex and nuanced than simply the provision of a sought‐after injectable opioid. As Ellefsen et al. [36] note, ‘[p]atient satisfaction with prescribed medical heroin cannot be detached from the setting in which [heroin assisted treatment] is provided and the way clinicians provide it’. Participant reports of their early experiences of hydromorphone varied, with heroin invariably functioning as an interpretative lens and comparative benchmark. The absence of heroin's ‘taste’, noted by a number of FOpIT participants, has similarly been reported by participants on previous SIOT trials that used pharmaceutical heroin [37]. Given hydromorphone is rarely found in Australia's illicit drug market, participants were unable to compare the effects of the study medication with any previous, non‐clinical experiences.

We also need to recognise that with the co‐prescription of methadone (or buprenorphine), participants may have struggled to disentangle the effects of the two drugs—something participants themselves observed anecdotally regarding the ‘blocking’ effects of methadone. Given methadone is considered an opioid agonist, the use of the term ‘blocking’ may seem erroneous here. However, it is well recognised among those with lived experience, and in the treatment literature, that methadone at sufficient doses can minimise, even negate, the effects of heroin and other opioids, such as hydromorphone. In a recent study examining the analgesic effectiveness of hydromorphone among hospitalised individuals on methadone treatment, the authors concluded that ‘methadone‐maintained individuals are highly *insensitive* to the analgesic effects of high‐dose IV hydromorphone’ ([38] emphasis added). On the other hand, the co‐prescription of buprenorphine and hydromorphone might reasonably seem more likely to present problems, given the potential for precipated withdrawal associated with the former. However, as noted earlier, neither Simon nor Ben, the only two participants on buprenorphine formulations, reported any adverse experiences, with both describing their early experiences of hydromorphone favourably. Finally, given the mandatory co‐prescription of methadone, another distinguishing feature of the trial, it is possible that FOpIT was the first time some participants had received a regular dose of methadone—an experience previously reported by participants of a Canadian trial [15].

Here, an argument posed by critical drug scholars warrants consideration [39]. Positing that no ‘pure’ pharmaceutical object precedes its socialisation, they contend that instead of explaining the effects of hydromorphone solely in terms of chemical determinism—as fixed, universal, and pre‐determined—we should understand them as ‘something in the making’, under ‘negotiation’, and inextricably contextualised [40]. As Hardon [29] argues, ‘pharmaceutical action is not reducible to the chemical properties of pharmaceuticals but is articulated, elicited, and informed within a meshwork of experimental, regulator, and care settings’. For most participants the practice of injecting was an important actor in the ‘making up’ of their hydromorphone experience. In the absence of injecting street heroin, injectable treatment worked both literally and symbolically to satisfy participants that they had had ‘their shot’, even among some who injected intramuscally. FOpIT participants also described the contingent, situated nature of pharmaceutical effects when observing how different settings (such as clinic and home) or the presence or absence of certain practices (such as smoking or listening to music) reconfigured their experience of hydromorphone. Finally, we note the therapeutic and pragmatic potential of pleasure in treatment [41]. Relative to standard OAT, SIOT recognises the importance of not only affording a more rewarding medication or pharmacotherapy [12] but a route of administration that is deeply meaningful for some—one that need not necessarily be foregone as a condition of treatment [42].

Mayer et al. [7] draw our attention to the role of the ‘structural vulnerabilities’ that inevitably shape people's sense of agency and decision‐making regarding SIOT, with the latter necessarily embedded within its own broader medical and political contexts. Within the lives of most participants, drug treatment with its attendant stigmatisation and marginalisation [43], has played a defining role. Virginia Berridge's [13] notion of ‘futurizing history’ draws our attention to the critical role of history in understanding and shaping the possible future(s) of addiction and its treatment, both for the individual (such as a FOpIT participant) and the collective (such as Australian drug treatment policy). Importantly, attending to the history of drug treatment alerts us to ‘the wolf of drug control dressed in the sheep's clothing of medicine’ [15], reminding us how ‘notions of “care” for undesirable Others [can] very quickly descend into techniques of control’ [44].

We recognise that the design and setting of the study may have unintentionally affected our findings. The significance of FOpIT being the first, long‐awaited trial of short‐acting, injectable opioid treatment in Australia, coupled with its newness in the local treatment context, may have contributed to the relatively high motivation reported among participants to achieve ‘positive’ treatment outcomes. It is also seems likely that the novelty of hydromorphone in the Australian context (as both an injectable treatment and diverted street drug) will have influenced participant experiences and accounts of the medication. These factors may limit the degree to which our findings are generalisable.

## 5 Conclusion

Both for those seeking and delivering treatment, the period of initiation is a crucial one, potentially setting the stage for and shaping subsequent treatment trajectories. In this sense, understanding the complexities of why and how people initiate SIOT has clear implications for clinical practice, including the potential realisation of improved therapeutic and clinical outcomes. How FOpIT participants accounted for their initial engagement in SIOT and for the effects of a new treatment medication revealed a diversity of motivations, hopes, goals, and effects. The extent to which these are carried through to treatment outcomes is the subject of ongoing research.

## Author Contributions

Each author certifies that their contribution to this work meets the standards of the International Committee of Medical Journal Editors.

## Conflicts of Interest

J.R., V.B., J.B., N.E., M.J., A.R., D.M.R., C.R., K.J.S., C.T.: none declared. N.L. has received funding for undertaking research studies with buprenorphine products from Camurus AB and Indivior and received honoraria from Camurus AB for a conference presentation. W.B. has received honoraria from Camurus, Indivior, Takeda and Mundipharma, none related to the treatment or study being described in this paper. J.S. has worked with pharmaceutical and technology companies to investigate new or improved medications for reducing opioid related harm: none is related to the treatment or study being described in this paper. A.J.D. is employed by a local health district which has received funds from Camurus AB and Indivior to conduct clinical research studies on long acting injectable buprenorphine, unrelated to the treatment or study described in this paper. Several of the authors (J.B., A.J.D., J.S., N.L., W.B., E.O.‐J. and C.R.) have each been, at different times, clinicians directly providing SIOT, and been active in seeking clinical funding support for SIOT. M.J. is the Director of the Sydney, Medically Supervised Injecting Centre. Many authors (J.B., A.J.D., J.S., N.E., C.R., D.M.R., W.B., N.L. and E.O.‐J.) have been providing opioid agonist treatments more broadly for a number of years.

## Data Availability

Research data are not shared.
